# Flexible Tactile Sensor Based on Patterned Ag-Nanofiber Electrodes through Electrospinning

**DOI:** 10.3390/s21072413

**Published:** 2021-03-31

**Authors:** Mengxiao Chen, Zhe Wang, Yu Zheng, Qichong Zhang, Bing He, Jiao Yang, Miao Qi, Lei Wei

**Affiliations:** 1 School of Electrical and Electronic Engineering, Nanyang Technological University, 50 Nanyang Avenue, Singapore 639798, Singapore; chenmx@ntu.edu.sg (M.C.); zwang030@e.ntu.edu.sg (Z.W.); yzheng018@e.ntu.edu.sg (Y.Z.); zhangqc@ntu.edu.sg (Q.Z.); bing.he@ntu.edu.sg (B.H.); ya0004ao@e.ntu.edu.sg (J.Y.); miao001@e.ntu.edu.sg (M.Q.); 2 CINTRA CNRS/NTU/THALES, UMI3288, Research Techno Plaza, Nanyang Technological University, 50 Nanyang Drive, Singapore 637553, Singapore; 3 The Photonics Institute, Nanyang Technological University, 50 Nanyang Avenue, Singapore 639798, Singapore

**Keywords:** electrospinning, Ag nanofibers, pressure mapping, TENG pressure sensors, flexible electrodes

## Abstract

The growing demand for intelligent equipment has greatly inspired the development of flexible devices. Thus, disparate flexible multifunctional devices, including pressure sensitive flexible/stretchable displays, have drawn worldwide research attention. Electrodes maintaining conductivity and mechanical strength against deformations are indispensable components in all prospective applications. In this work, a flexible pressure mapping sensor array is developed based on patterned Ag-nanofibers (Ag-NFs) electrode through electrospinning and lithography. The metallic Ag layer is sputtered onto the electrospinning polyvinyl alcohol (PVA) NFs. A uniform and super conductive electrode layer with outstanding mechanical performance is thus formed after dissolving PVA. Followed by the traditional lithography method, a patterned electrode array (4 × 4 sensors) is obtained. Based on the newly developed triboelectric nanogenerator (TENG) technology, a flexible pressure-mapping sensor with excellent stability towards bending deformations is further demonstrated. Moreover, a letter “Z” is successfully visualized by this pressure sensor array, encouraging more human–machine interactive implementations, such as multi-functional tactile screens.

## 1. Introduction

Flexible devices, such as flexible displays, flexible sensors, and flexible optoelectronic devices, are vital important for future intelligent electronic systems [1]. To develop flexible devices, the flexible/stretchable electrode is an indispensable component. Recent studies have reported various solutions on flexible/stretchable electrodes, including traditional carbon-based electrodes [2,3,4,5,6], indium tin oxide (ITO) films [7], ionic conductors [8], and conducting polymers [9,10]. However, the limited flexibility of ITO electrodes and carbon-based electrodes, instability of ionic conductors, and the low conductivity of conducting polymers largely hinder their implementations. Thus, new solutions are highly demanded. Conductive metallic nanofibers (NFs) have drawn great attention to act as electrodes of flexible and stretchable electronics since firstly proposed [11]. They exhibit excellent performance in both mechanical property and conductivity [12]. So far, conductive metallic NFs (e.g., Ag NFs [13], Cu NFs [14]) based electrodes have been reported for numerous devices, such as touch-screen panels [15] and wireless electronics [16], which maintain good stability and super conductivity against mechanical strains, forecasting a series of novel applications based on this promising electrode design.

The emerging triboelectric nanogenerator (TENG) technology utilizing the triboelectrification effect and electrostatic induction has enabled widespread applications in self-powered sensors [17]. It generates electricity from physically manipulating two materials with opposing surface charges. Simply driven by walking or running, the output electricity of TENGs is enough to power sensors, microcontrollers, memories, arithmetic logic units, displays, and even wireless transmitters [18,19]. This gives the opportunity to develop novel sensor devices towards self-powered electronics.

Here, we demonstrate a flexible tactile sensor, combining the Ag NFs electrode array with the single electrode TENG technology [20]. An Ag NFs electrode layer was fabricated through electrospinning process [21,22] and then transferred to the flexible polyethylene terephthalate (PET) substrate after dissolving the water soluble polyvinyl alcohol (PVA) supporting core. By adopting lithography approaches and wet etching, the transferred Ag NFs layer was further manufactured into a 4 × 4 electrodes array serving as sensing layer. After packaging with Polydimethylsiloxane (PDMS), each square electrode pixel acted as a single electrode TENG to detect mechanical stimulations from both static and dynamic processes by converting ambient mechanical energy into electric energy. The reconstructed pressure map successfully revealed a “Z” shaped stamp used in the experimentation after analyzing electric signals from all pixels, in which pixels contacted with the stamp ridge area show higher outputs. Furthermore, the device exhibited good repeatability and stability, with almost unchanged output signal after long-term measurement and 2000 bending cycles. Prospectively, tactile sensing and pressure displays towards human machine interacting applications could be further explored.

## 2. Materials and Methods

The proposed pressure mapping sensor array was mainly based on the patterned Ag NFs electrode on a flexible substrate. Firstly, a conductive NFs electrode layer was fabricated through electrospinning and magnetron sputtering. Then it was transferred onto a flexible PET film. Followed by UV lithography approaches, the electrode layer was manufactured into square pixels to form an array. The pressure mapping sensor array was obtained after packaged with PDMS.

**Figure 1:**
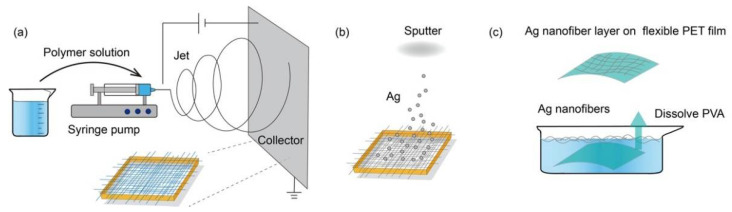
Schematic of the fabrication process of the Ag nanofibers (NFs) layer on the flexible polyethylene terephthalate (PET) film substrate. (**a**) Sketch diagram of the electrospinning of polyvinyl alcohol (PVA) NFs. (**b**) The simplified process of depositing Ag layer onto the surface of collected PVA NFs. (**c**) Diagram of dissolving the supporting PVA NFs core and transferring Ag NFs onto the flexible PET film surface.

**Figure 2:**
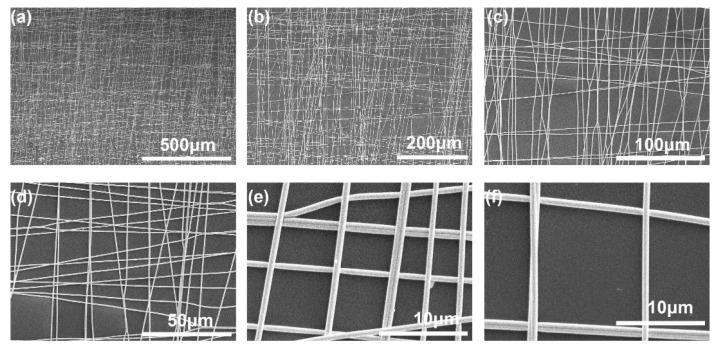
SEM images of the bidirectional Ag-PVA nanofibers, revealing a uniform nanofiber membrane. (**a**–**d**) The SEM images under different magnification to show the uniform distribution of the bidirectional NFs membrane. (**e**,**f**) Enlarged SEM images to demonstrate the individual NFs and their cross connections.

Figure 1 shows the fabrication process and the transferring process of the Ag NFs electrode membrane. PVA (molecular weight ~80,000) aqueous solution with a concentration of 10 wt% was firstly prepared. The mixture was stirred for 24–36 h to obtain the homogeneous solution. Then, the PVA aqueous solution was filled into a 10 mL syringe with a 22 G needle for electrospinning. The speed of the injection pump was set at 0.4 mL/h, the positive voltage was set at 9 kV, and the negative voltage was set at −4 kV. Thus, a total voltage of 13 kV was applied between the needle and the substrate. To get a layer of PVA NFs oriented in two orthogonal directions, a rectangular holder with dimensions of 7.5 cm × 7.5 cm × 5 cm was utilized to collect the electrospun fibers. The humidity was kept at 25%, and it took 20 min to collect enough PVA NFs with an average fiber diameter of 400 nm. It worth noting that low humidity contributes to the formation of long PVA fibers, as well as their smooth surface, due to the water solubility of PVA. The second step was sputtering the Ag layer onto the surface of the collected PVA NFs membrane (Figure 1b). The magnetron sputtering power was 100 W under the Ar atmosphere with a gas pressure 4 m Torr. The sputtering time was 15 min to obtain an Ag layer of 250 nm. After coated with Ag layer, the diameter of the fibers was about 650 nm as shown in Figure 2e,f. The fiber orientation of electrospinning PVA NFs could be controlled by the shape of the collectors. Unidirectional, bidirectional, tridirectional, and random directional fiber orientations have been reported in previous works [15]. The transparence of the Ag NFs electrode layer is closely related to the thickness of PVA NFs membrane, which could be controlled by the spinning time. Figure 2a–d reveals a uniform distribution of the bidirectional NFs coated with Ag layer.

The next transferring process was dissolving PVA in water and relocating Ag NFs on flexible PET substrate. The nanofiber membrane was firstly placed on the surface of the water and a 125 μm-thick PET film was placed below. When PVA NFs were fully dissolved, the PET film was lifted from the water to hold the NFs of Ag shell, as illustrated in Figure 1c. Then it was dried under hot air with temperate of ~50 °C and flow rate of ~2.5 m^3^/min to enhance the adhesion. Then the Ag NFs covered PET substrate was immersed into water and dried under hot air, repeated for three times, so the adhesion between Ag NFs, and the PET substrate, was greatly enhanced. Then, the PET film with Ag NFs electrode layer on the surface was baked for 20 min in a 180–220 °C oven to release the inner stress of Ag NFs. To ensure the adhesion was strengthened to the greatest extent, the final step was to immerse the Ag NFs covered PET sheet in methanol and dry it one more time. In this repeated transferring and drying process, the cross connection of nanofibers was enhanced to guarantee the conductivity and the mechanical property under deformations. Through optimizing the humidity of electrospinning process and adhesion force between Ag NFs network and substrate, 97% reproducibility of the Ag NFs layer was achieved.

**Figure 3:**
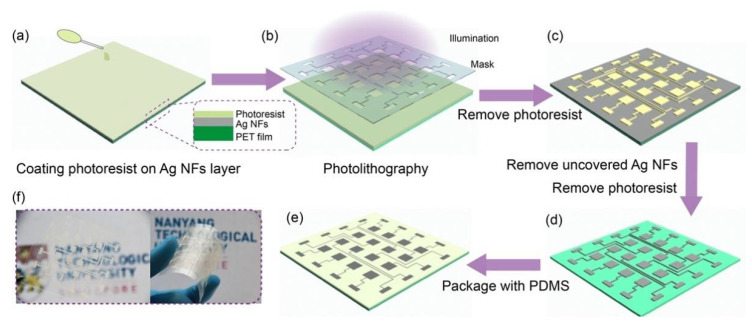
Flow chart of the photolithography process to fabricate the patterned Ag NFs electrode and to package as the single electrode triboelectric nanogenerator (TENG) sensor array device. (**a**) Spin coating photoresist on Ag NFs layer. (**b**) The substrate with photoresist exposed under UV light illumination with designed mask. (**c**) Developing the pattern and removing the photoresist. (**d**) Removing the uncovered area of Ag NFs layer and then removing the photoresist covering on top of patterned Ag NFs, to obtain a patterned Ag NFs electrode array on the PET substrate. (**e**) Packaging the electrode array with Polydimethylsiloxane (PDMS). (**f**) The optical photos of patterned Ag NFs electrode array on PET film, indicating its flexibility and transparence.

To fabricate the Ag NFs conductive layer into a patterned electrode array, a traditional UV lithography method was employed. Figure 3 illustrates the technological flow chart of fabricating the patterned electrode. Firstly, the photoresist NR9 1500PY was spin-coated onto the Ag NFs layer with 4000 rpm for 30 s (Figure 3a). Then it was pre-baked for 60 s at 150 °C and illuminated for 30 s under the designed mask (Figure 3b). After post-baking for 60 s at 100 °C, unilluminated photoresist was removed by developing for 30 s (Figure 3c). Next, the uncovered Ag NFs were etched and removed in HNO_3_ (5 M) for 30 s. Moreover, the remaining photoresist on top of the patterned Ag NFs was dissolved in acetone (Figure 3d). Thus, a 4 × 4 Ag NFs electrode array with a pixel area of 4 mm × 4 mm was successfully obtained. After packaged with PDMS layer (SYLGARD184 Dow Corning, 10:1 mixture ratio by weight), the pressure sensor array based on patterned Ag NFs electrode was completed.

## 3. Results

The as fabricated Ag NFs based sensor array device works in single electrode TENG mode and each square electrode acts as an individual sensor. After signals from all the 4 × 4 electrodes are collected, a signal map could be generated correspondingly to reveal pressure information applied on this sensor panel.

### 3.1. Test Process and Working Mechanism of the Pressure Mapping Sensor

**Figure 4:**
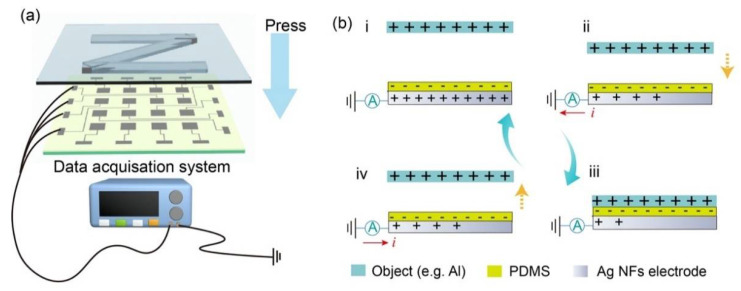
The pressure mapping test and the working principle. (**a**) Each pixel is connected to the data acquisition equipment through leading wire, and sensing data are collected from electrometers. When the stamp is pressed on to the pixel array, the output signal from pixels contacting the stamp ridges increases to differentiate the stamp shape. (**b**) Description of the working mechanism of the single electrode TENG pressure sensor. (**i**) The original state where the object is away from the sensor, the sensor is at the electrostatic equilibrium state. (**ii**) When the object with positive charges gets close to the sensor, to keep the electrostatic equilibrium state, the positive charges at the electrode interface decreases, resulting in a current from the sensor electrode to the external circuit until fully contacted. (**iii**) When the object is fully contacted with the sensor surface, the sensor reaches another static equilibrium state. (**iv**) When the object moves away from the sensor, an opposite process of (**ii**) happens, to maintain the electrostatic equilibrium state with positive charges being away from the PDMS surface, positive charges move from external circuit towards the sensor electrode.

Figure 4a illustrates the data acquisition process. Data from each of the 16 pixels was collected through the data acquisition system, which was composed of a Keithley 6517B electrometer and a Stanford 570 current preamplifier. The open-circuit voltage and short-circuit transferring charge were measured by Keithley 6517B electrometer and short-circuit current was measured by Stanford 570. The “Z” shaped stamp was fixed on the linear motor and each pixel from the array was connected to the data acquisition equipment. The real-time pressure was recorded by the force gauge. After the motor started working, the stamp moved towards the mounted device. When the “Z” zone from the stamp contacted the device, signals were generated from the corresponding pixels. While no signal was generated from the other untouched pixels. The working mechanism of the single electrode TENG is described in Figure 4b. Figure 4b(i) indicates the original state where the object with positive charges on its surface (e.g., aluminum plate) is away from the sensor. Moreover, the sensor is at the electrostatic equilibrium state. When the object with positive charges gets close to the sensor, to keep the electrostatic equilibrium state, the positive charges in the sensor electrode transfer to the earth through the external circuit decreases, resulting in a current until the object fully contact the sensor. The direction of the current is “+” (Figure 4b(ii)). When the object fully contacts the sensor surface, the sensor reaches a new static equilibrium state as shown in Figure 4b(iii). When the object moves away from the sensor, an opposite process of (ii) happens. As positive charges on the object surface moving away from the PDMS surface, positive charges move from earth towards the Ag NFs through the external circuit. The direction of the current changes to “-”. Thus, when a stamp with a certain shaped is pressed onto the sensor array, the corresponding output signals from the 16 pixels would be generated.

### 3.2. Pressure Mapping Result of a Letter “Z” on the Patterned Ag-NFs Based Sensor Array

**Figure 5:**
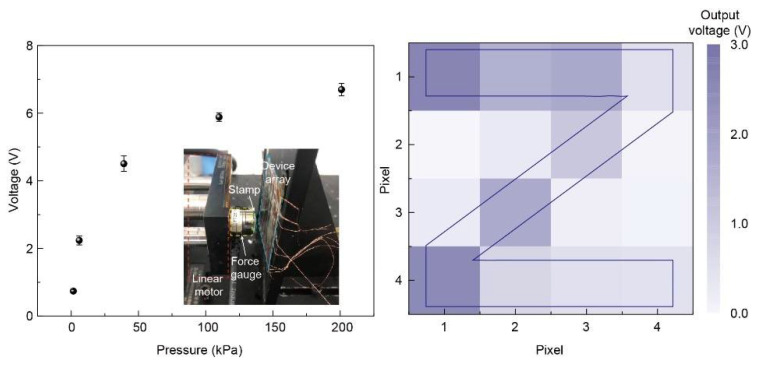
**Left**: output voltage of a single pixel as a function of the applied pressure. The inset shows the experimental setup to apply and measure the pressure sensing. **Right**: the pressure mapping result of the “Z” shaped stamp with output voltage signals, in which pixels contacted with the stamp ridge area show a higher output.

The dependence of output voltage on the applied pressure for the patterned Ag-NFs based sensor array was investigated firstly. As shown in Figure 5 (left panel), the output voltage increases with the applied pressure, demonstrating the pressure sensing range from 1.6 kPa to 200 kPa. Under small pressure condition, the output voltage increases dramatically, which is attribute to the fact that the close contact between the stamp and the device starting to form. As the pressure further increases, the saturation of the output voltage occurred, indicating that the surfaces have been fully contacted. A “Z” shaped stamp was used to test the patterned Ag-NFs based sensor array. The output voltages were collected to analyze the pressure distribution while the stamp was pressed onto and released from the sensor panel periodically driven by the linear motor. Figure 5 (right panel) demonstrates the visualization results of voltage values corresponding to the 16 pixels. The letter “Z” was clearly demonstrated, which indicates the feasibility of the proposed working principle and device designing. The variation of the output voltage from the contacted pixels is due to the angle between the stamp and device array in vertical direction. Based on this result, more precise device applications could be further explored.

### 3.3. Repeatability and Stability of the Sensor Array

**Figure 6:**
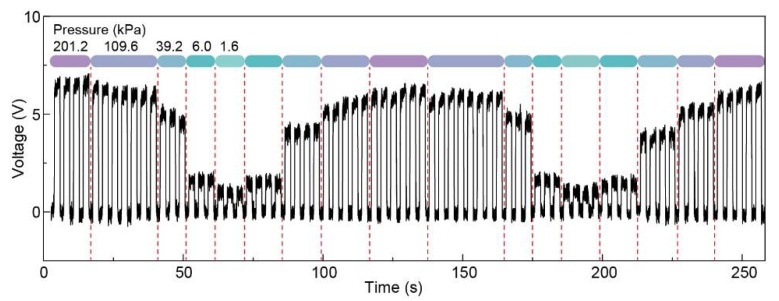
Output voltage under various pressures to show the repeatability of pressure sensing measurement for the patterned Ag NFs electrode based pressure sensor array.

Though only voltage signals from the 16 pixels were utilized to visualize the pressure map, all the electric signals including voltage, transferring charge, and current were collected to illustrate the repeatability and stability of the sensor array. Pressures with 1.6, 6, 39.2, 109.6, and 201.2 kPa were applied onto the device, and carried out four cycles. As shown in Figure 6, for one certain pressure, there is no obvious difference in the voltage outs from different cycles, indicating excellent stability and repeatability.

**Figure 7:**
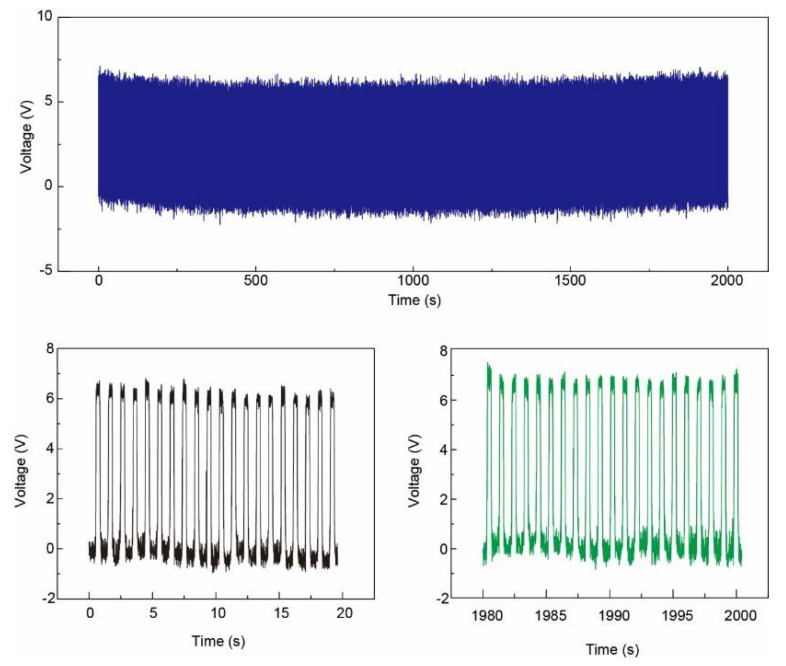
**Top**: output voltage for 2000 s (~2000 circles) to show the stability of the patterned Ag NFs electrode based pressure sensor array. **Bottom**: output voltage of the beginning and the end of the test. The output voltage from one fixed pixel kept between 6–7 V.

Figure 7 demonstrates the output voltage from one pixel continuously working for 2000 s (~10,000 circles, contact–separate controlled by the linear motor) to show the stability of the patterned Ag NFs electrode based pressure sensor array. The output voltage from this pixel kept between 6–7 V.

**Figure 8:**
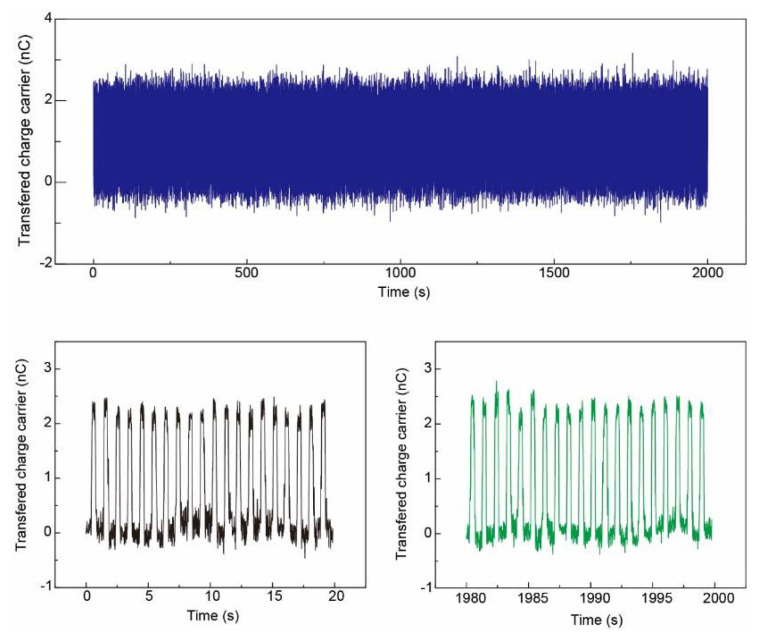
**Top**: transferring charge amount for 2000 s (~2000 cycles) to show the stability of the Ag NFs electrode based pressure sensor array. **Bottom**: output voltage of the beginning and the end of the test. The transferring charge amount in one working cycle maintained at around 2.5 nC.

Figure 8 demonstrates the transferring charge amount from one pixel for 2000 s (~10,000 cycles, contact–separate controlled by the linear motor) to show the stability of the Ag NFs electrode based pressure sensor array. The transferring charge amount in one working cycle maintained at around 2.5 nC.

**Figure 9:**
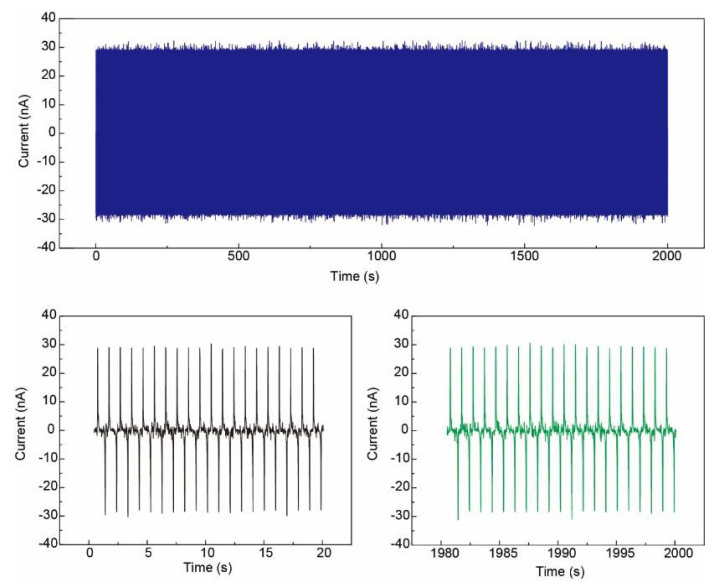
**Top**: output current for 2000 s (~2000 cycles) to show the stability of the Ag NFs electrode based pressure sensor array. **Bottom**: output voltage of the beginning and the end of the test. The output current of one contact–separate cycle kept about 30 nA.

Figure 9 demonstrates the output current from one pixel for 2000 s (~10,000 cycles, contact–separate controlled by the linear motor) to show the stability of the Ag NFs electrode based pressure sensor array. The output current of one contact–separate cycle kept at about 30 nA.

**Figure 10:**
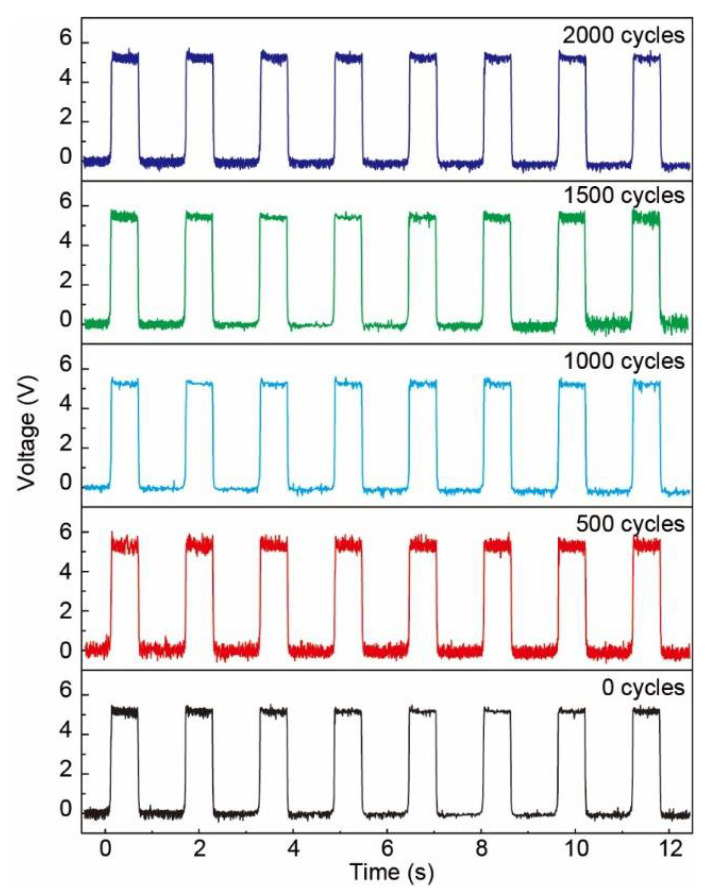
Output voltage for 2000 bending (bending angle: ~30°) cycles to show the mechanical stability of the Ag NFs electrode based pressure sensor array. The output voltage kept at 5–6 V in the bending test.

The bending stability of the Ag NFs electrode based flexible pressure sensor array was tested as shown in Figure 10. Output voltage from one fixed pixel during 2000 bending cycles (bending angle: ~30°) was recorded to show the mechanical stability of the Ag NFs electrode based pressure sensor array. The output voltage kept at 5–6 V in the bending test, indicating the excellent stability of this Ag NFs electrode towards bending deformations. Thus, applications, such as tactile sensing and pressure displays, which need high levels of stability, could be further developed.

## 4. Discussion

The results presented here demonstrate a simple method to fabricate a flexible tactile sensor array based on the Ag NFs network. The simple sandwiched device structure will facilitate the integration with large-scale multifunctional sensing systems. The Ag NFs offered outstanding performance in both conductivity and mechanical strength compared to the traditional carbon based electrode, ionic conductor based electrode, and conducting polymers. Such a straightforward method for patterning the Ag NFs is of great portability for other device designs based on the metallic nanowire networks. More efforts could be expected to further improve the performance. For example, modern microfabrication processing method could be utilized to minimize the pixel size, achieving more precise sensing. The substrate could also be further modified to be stretchable substrates, and the electronegativity of the packaging layer could be optimized to keep more electrons on its surface for obtaining higher electrical outputs. Moreover, human–machine interactive implementations, such as multi-functional tactile screens, could thus be expected.

## 5. Conclusions

In summary, we developed a flexible tactile sensor array based on the patterned Ag NFs and the single electrode TENG working principle for pressure mapping. The Ag NFs were obtained by coating Ag layer onto the electrospinning PVA NFs, and then removing the supporting PVA core fiber in water. After transferring the Ag NFs layer to the flexible PET substrate, the electrode array with 16 pixels was patterned via photolithography. The tactile sensing properties of the as-fabricated device was systematically investigated, showing good mechanical stability with almost unchanged output voltage after 2000 bending cycles. A letter “Z” was successfully recognized by the proposed sensor array, indicating the great potential in high-resolution self-powered flexible pressure mapping devices.

## Data Availability

Not applicable.
